# Supplementary material for: Association between oral microbiome and five types of respiratory infections: a two-sample Mendelian randomization study in east Asian population
Source: Front Microbiol. 2024 Apr 10;15:1392473. doi: 10.3389/fmicb.2024.1392473 (PMC11039966; doi:10.3389/fmicb.2024.1392473)
Supplement: Supplementary file 1 [file Data_Sheet_1.zip › Supplementary Materials/MR plots of saliva/Pneumonia/s__Actinomyces_oris_D_mgs_691/leave_one_out.pdf]

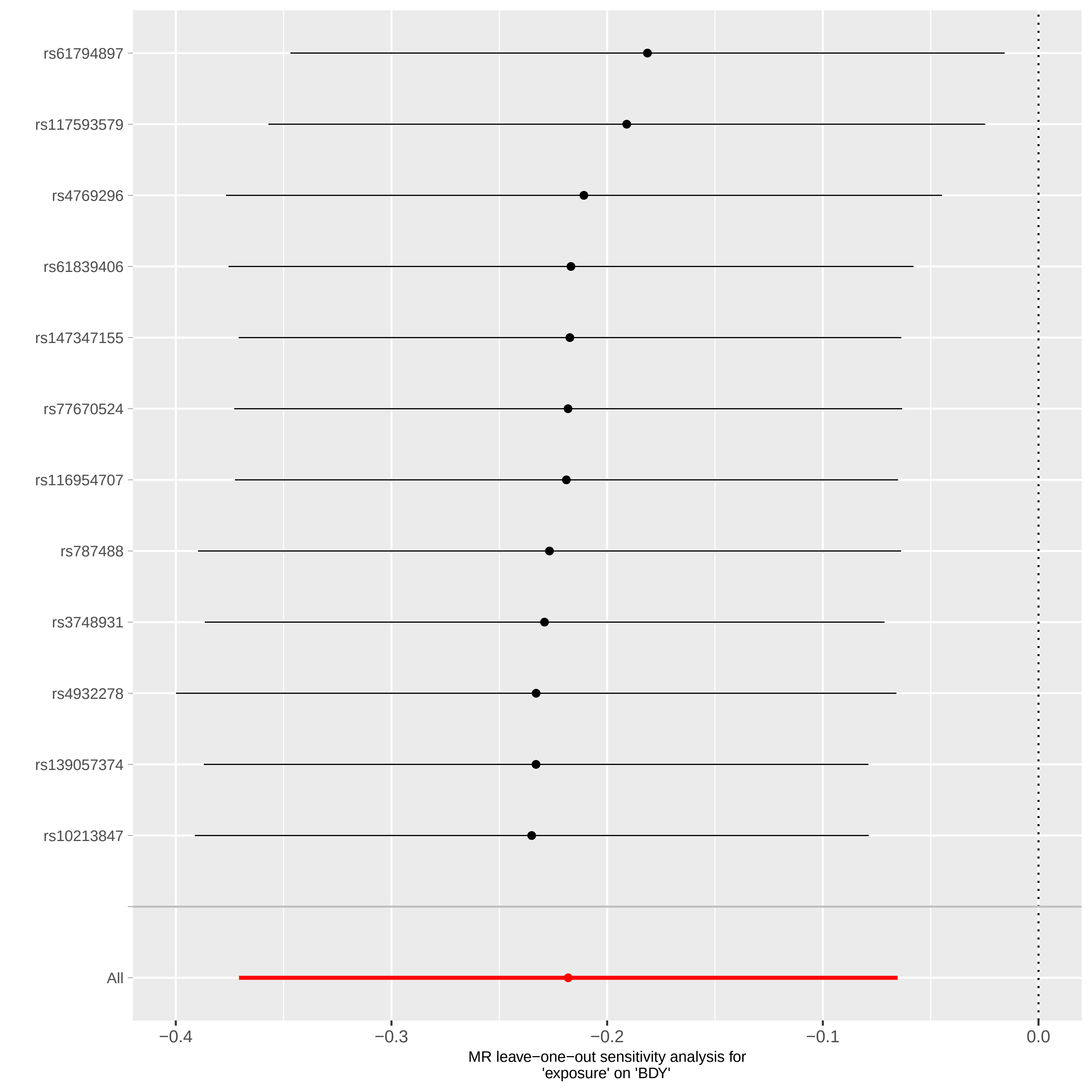

rs61794897
rs117593579
rs4769296
rs61839406
rs147347155
rs77670524
rs116954707
rs787488
rs3748931
rs4932278
rs139057374
rs10213847
All
−0.4
−0.3
−0.2
−0.1
0.0
MR leave−one−out sensitivity analysis for
'exposure' on 'BDY'
